# Supplementary material for: Selective lipid recruitment by an archaeal DPANN symbiont from its host
Source: Nat Commun. 2024 Apr 22;15:3405. doi: 10.1038/s41467-024-47750-2 (PMC11035636; doi:10.1038/s41467-024-47750-2)
Supplement: Supplementary file 3 — Description of Additional Supplementary Files [file 41467_2024_47750_MOESM3_ESM.pdf]

Description of Additional Supplementary Files

File Name: Supplementary Data 1
Description: FISH based cell size and shape statistics. Channel 1 (C1) represents *Hrr. lacusprofundi* 16s rRNA positive cells, Channel 2 (C2) represents *Ca.* Nha. antarcticus 16s rRNA positive cells.

File Name: Supplementary Data 2
Description: Annotation table of the *Halorubrum lacusprofundi* R1S1 genome

File Name: Supplementary Data 3
Description: Annotation table of lipid biosynthesis genes identified within the *Halorubrum lacusprofundi* R1S1 genome

File Name: Supplementary Data 4
Description: Annotation table of the *Ca.* Nha. antarcticus genome

File Name: Supplementary Data 5
Description: Annotation table of lipid biosynthesis genes identified within the *Ca.* Nha. antarcticus genome
