# Supplementary material for: Selective lipid recruitment by an archaeal DPANN symbiont from its host
Source: Nat Commun. 2024 Apr 22;15:3405. doi: 10.1038/s41467-024-47750-2 (PMC11035636; doi:10.1038/s41467-024-47750-2)
Supplement: Supplementary file 9 — Reporting Summary [file 41467_2024_47750_MOESM9_ESM.pdf]

nature portfolio

Corresponding author(s): Su Ding, Joshua N. Hamm

Last updated by author(s): Mar 22, 2024

Reporting Summary

Nature Portfolio wishes to improve the reproducibility of the work that we publish. This form provides structure for consistency and transparency in reporting. For further information on Nature Portfolio policies, see our Editorial Policies and the Editorial Policy Checklist.

Statistics

For all statistical analyses, confirm that the following items are present in the figure legend, table legend, main text, or Methods section.

n/a	Confirmed	
☐	☒	The exact sample size (*n*) for each experimental group/condition, given as a discrete number and unit of measurement
☐	☒	A statement on whether measurements were taken from distinct samples or whether the same sample was measured repeatedly
☐	☒	The statistical test(s) used AND whether they are one- or two-sided<br>*Only common tests should be described solely by name; describe more complex techniques in the Methods section.*
☐	☒	A description of all covariates tested
☐	☒	A description of any assumptions or corrections, such as tests of normality and adjustment for multiple comparisons
☐	☒	A full description of the statistical parameters including central tendency (e.g. means) or other basic estimates (e.g. regression coefficient) AND variation (e.g. standard deviation) or associated estimates of uncertainty (e.g. confidence intervals)
☐	☒	For null hypothesis testing, the test statistic (e.g. *F*, *t*, *r*) with confidence intervals, effect sizes, degrees of freedom and *P* value noted<br>*Give P values as exact values whenever suitable.*
☒	☐	For Bayesian analysis, information on the choice of priors and Markov chain Monte Carlo settings
☒	☐	For hierarchical and complex designs, identification of the appropriate level for tests and full reporting of outcomes
☒	☐	Estimates of effect sizes (e.g. Cohen's *d*, Pearson's *r*), indicating how they were calculated

Our web collection on statistics for biologists contains articles on many of the points above.

Software and code

Policy information about availability of computer code

Data collection	MZmine 2.5.3, https://github.com/mzmine/mzmine2/releases<br>Xcalibur 4.1, https://www.thermofisher.com/<br>GNPS platform, https://gnps.ucsd.edu/ProteoSAFe/static/gnps-splash.jsp<br>Zeiss Zen Blue, https://www.zeiss.com/microscopy/en/products/software/zeiss-zen.html<br>Data was collected using proprietary software of the instruments used to collect and collection procedure is described within the methods.<br>Additional workflows and custom scripts are provided via our zenodo repository [https://doi.org/10.5281/zenodo.10258365].
Data analysis	GNPS platform, https://gnps.ucsd.edu/ProteoSAFe/static/gnps-splash.jsp<br>Xcalibur 4.1, https://www.thermofisher.com/<br>Cytoscape 3.9.0, https://cytoscape.org/download.html<br>Prokka v1.14, https://github.com/tseemann/prokka<br>trimmomatic v0.36, www.usadellab.org/cms/?page=trimmomatic<br>SPAdes v3.15.0, https://github.com/ablab/spades<br>quast v4.6.3, https://quast.sourceforge.net/<br>InterProScan v5.62-94.0, https://www.ebi.ac.uk/interpro/download/interproscan/<br>hmmsearch v3.1b2, http://hmmer.org/download.html<br>BLASTp v2.7.1, https://ftp.ncbi.nlm.nih.gov/blast/executables/blast+/LATEST/<br>Part of the data analysis was conducted using custom scripts written in Fiji (ImageJ), R, and Bash. All scripts and workflows are available via our repository on zenodo [https://doi.org/10.5281/zenodo.10258365].

For manuscripts utilizing custom algorithms or software that are central to the research but not yet described in published literature, software must be made available to editors and reviewers. We strongly encourage code deposition in a community repository (e.g. GitHub). See the Nature Portfolio guidelines for submitting code & software for further information.

Data

Policy information about availability of data

All manuscripts must include a data availability statement. This statement should provide the following information, where applicable:

- Accession codes, unique identifiers, or web links for publicly available datasets
- A description of any restrictions on data availability
- For clinical datasets or third party data, please ensure that the statement adheres to our policy

The processed data (.mgf and .csv) with the molecular network and detailed parameter settings used in this study are available at the GNPS platform under accession code [https://gnps.ucsd.edu/ProteoSAFe/status.jsp?task=c75ddcdd8d2e426e9d537ee1037a2b43]. The raw data of lipidome used in this study is available in MassIVE under accession code [https://doi.org/10.1101/2023.12.06.570485]. The Halorubrum lacusprofundi R1S1B genome used in this study has been deposited at DDBJ/ENA/GenBank under the accession JAXGGM000000000, BioProject: PRJNA1046704, BioSample: SAMN38507334. The version described in this paper is version JAXGGM010000000. Databases used for genome annotation can be found at: COGs 2020 update (https://ftp.ncbi.nlm.nih.gov/pub/COG/COG2020/data/), arCOGs 2018 (https://ftp.ncbi.nlm.nih.gov/pub/wolf/COGs/arCOG/) KEGG 2021 (https://www.kegg.jp/kegg/download/), TIGRFAMs 15.0 (https://ftp.ncbi.nlm.nih.gov/hmm/TIGRFAMs/release_15.0/), CAZy Database 7 (http://www.cazy.org/spip.php?rubrique59), Hydrogenase Database 2020 (https://services.birc.au.dk/hyddb/browser/), NCBI_nr 2021 (https://ftp.ncbi.nlm.nih.gov/blast/db/), Transporter Classification Database 2021 (https://www.tcdb.org/download.php). The source data for all the figures are provided either in the supplementary tables or in the extended dataset in our data repository on zenodo [https://doi.org/ 10.5281/zenodo.10851289].

Research involving human participants, their data, or biological material

Policy information about studies with human participants or human data. See also policy information about sex, gender (identity/presentation), and sexual orientation and race, ethnicity and racism.

Reporting on sex and gender	The study does not involve human participants
Reporting on race, ethnicity, or other socially relevant groupings	The study does not involve human participants
Population characteristics	The study does not involve human participants
Recruitment	The study does not involve human participants
Ethics oversight	The study does not involve human participants

Note that full information on the approval of the study protocol must also be provided in the manuscript.

Field-specific reporting

Please select the one below that is the best fit for your research. If you are not sure, read the appropriate sections before making your selection.

☒ Life sciences ☐ Behavioural & social sciences ☐ Ecological, evolutionary & environmental sciences

For a reference copy of the document with all sections, see nature.com/documents/nr-reporting-summary-flat.pdf

Life sciences study design

All studies must disclose on these points even when the disclosure is negative.

Sample size	No statistical tests were conducted to assess appropriate sample size. Sample size for culture replicates were selected based on convention within the field (see Boyd et. al. 2010 https://doi.org/10.1007/s00792-010-0339-y, Elling et. al. 2014 https://doi.org/10.1016/j.gca.2014.07.005, and Feyhl-Buska et. al. 2016 https://doi.org/10.3389/fmicb.2016.01323). As all cultures used within the study are genetically identical the expected degree of variation between samples is low and more than 3 replicates is typically not necessary.
Data exclusions	No data were excluded
Replication	3 replicates for each condition within the study represent biological replicates to ensure differences were consistent across different incubations and statistical tests were conducted using all replicates to account for potential batch variation. Outside of data presented in the manuscript experiments have not been repeated due to technical difficulty and cost associated with carrying out the protocol. All replicates produced comparable results with low margins of error in all measurements.
Randomization	Randomization was not necessary for these experiments. The cultures used in the experiment are all genetically identical and as such there is no risk of genetic variation between cultures impacting results. To ensure all cultures were at identical stages of growth all replicates for both

conditions cultures were subcultured from the same starting culture and split into replicates immediately prior to the beginning of the experiment.

Blinding

Blinding was not necessary for these experiments. The experiment did not involve continued treatment of samples following the initial establishment of the cultures (which could not be done blind). Following this the experiment was limited to collecting samples at specific time points so no further bias could be introduced. However, the technician responsible for lipid extraction was not aware of which sample IDs corresponded to which samples and so the lipid extractions were carried out effectively blind.

Reporting for specific materials, systems and methods

We require information from authors about some types of materials, experimental systems and methods used in many studies. Here, indicate whether each material, system or method listed is relevant to your study. If you are not sure if a list item applies to your research, read the appropriate section before selecting a response.

Materials & experimental systems

n/a	Involved in the study
☒	☐ Antibodies
☒	☐ Eukaryotic cell lines
☒	☐ Palaeontology and archaeology
☒	☐ Animals and other organisms
☒	☐ Clinical data
☒	☐ Dual use research of concern
☒	☐ Plants

Methods

n/a	Involved in the study
☒	☐ ChIP-seq
☒	☐ Flow cytometry
☒	☐ MRI-based neuroimaging

Plants

Seed stocks

Report on the source of all seed stocks or other plant material used. If applicable, state the seed stock centre and catalogue number. If plant specimens were collected from the field, describe the collection location, date and sampling procedures.

Novel plant genotypes

Describe the methods by which all novel plant genotypes were produced. This includes those generated by transgenic approaches, gene editing, chemical/radiation-based mutagenesis and hybridization. For transgenic lines, describe the transformation method, the number of independent lines analyzed and the generation upon which experiments were performed. For gene-edited lines, describe the editor used, the endogenous sequence targeted for editing, the targeting guide RNA sequence (if applicable) and how the editor was applied.

Authentication

Describe any authentication procedures for each seed stock used or novel genotype generated. Describe any experiments used to assess the effect of a mutation and, where applicable, how potential secondary effects (e.g. second site T-DNA insertions, mosiacism, off-target gene editing) were examined.
